# Chronic Empyema Necessitans Perpetuated by ESBL *Klebsiella pneumoniae*

**DOI:** 10.1002/rcr2.70374

**Published:** 2025-10-08

**Authors:** Zhong Xhen Khor

**Affiliations:** ^1^ International Medical University Clinical Campus Department of Medicine Seremban Malaysia

**Keywords:** empyema necessitans, pleural, pleurocutaneous fistula, radiology, respiratory

## Abstract

A man with prior tuberculosis developed recurrent empyema necessitans, complicated by extended‐spectrum beta‐lactamase *Klebsiella pneumoniae* . Despite prolonged antimicrobials, serial imaging revealed a persistent pleurocutaneous fistula and pleural thickening. This case highlights that not all empyema necessitans is tuberculous, and delayed surgical intervention may necessitate morbid procedures such as pleural window.

## Graphical abstract

Empyema necessitans is not always tuberculous. Secondary bacterial superinfection, such as ESBL *Klebsiella pneumoniae* , may perpetuate chronic pleural infection. Early microbiological reassessment and timely surgical intervention are critical to avoid progression to refractory, morbid disease.

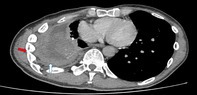

A 56‐year‐old man with prior pulmonary tuberculosis presented with recurrent right chest wall swelling and pus‐discharging wounds (Figure 1). Chest X rays showed right‐sided loculated homogeneous opacity, rib crowding, with volume loss, consistent with chronic empyema (Figure 2). Chest tomography revealed empyema thoracis with a pleurocutaneous fistula (Figure 3). As pleural fluid was GeneXpert‐positive for *Mycobacterium tuberculosis* (albeit at low titers), antitubercular therapy was initiated.

**FIGURE 1:**
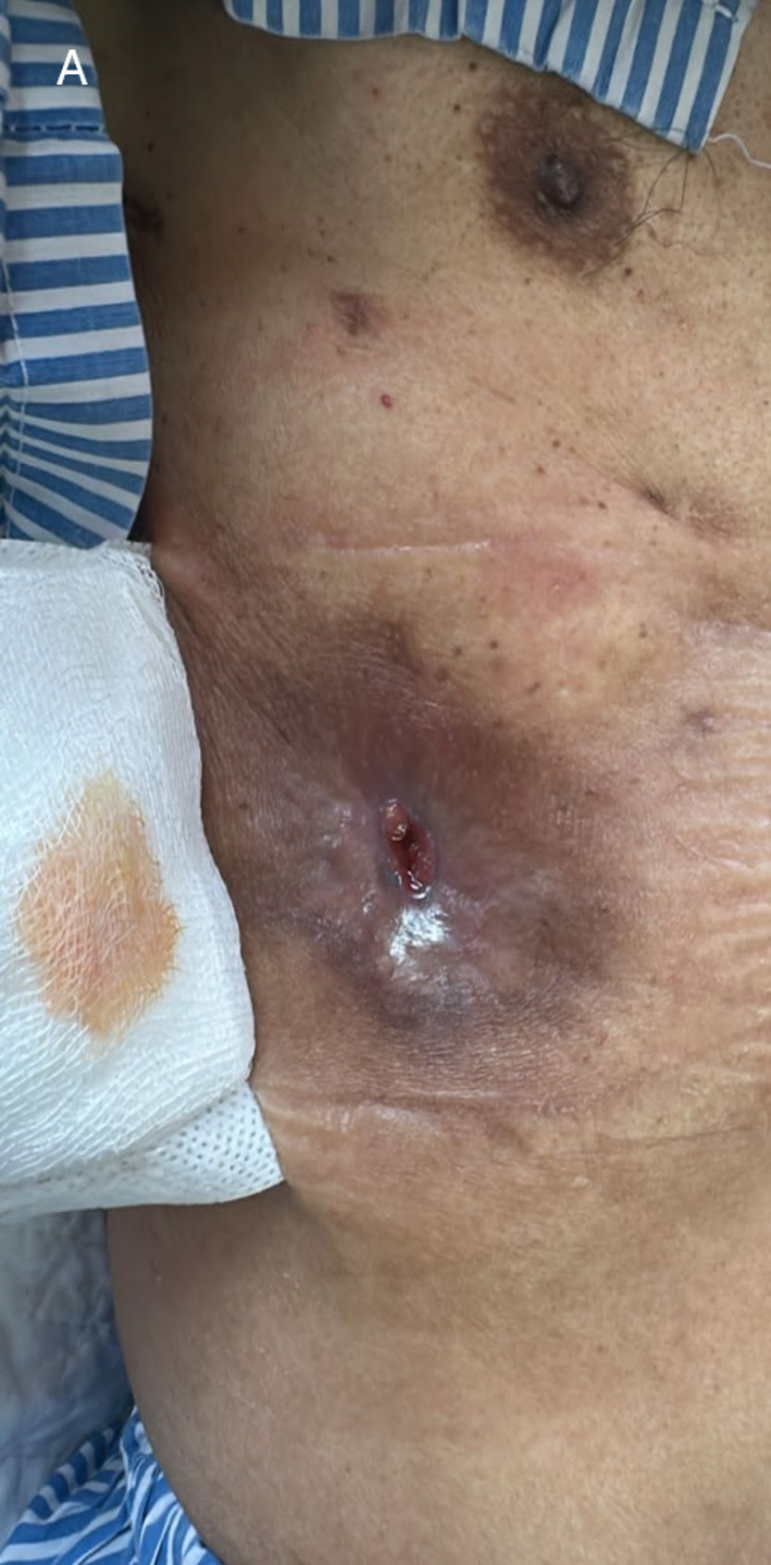
Pleurocutaneous fistula with overlying skin trophic changes and hyperpigmentation.

**FIGURE 2:**
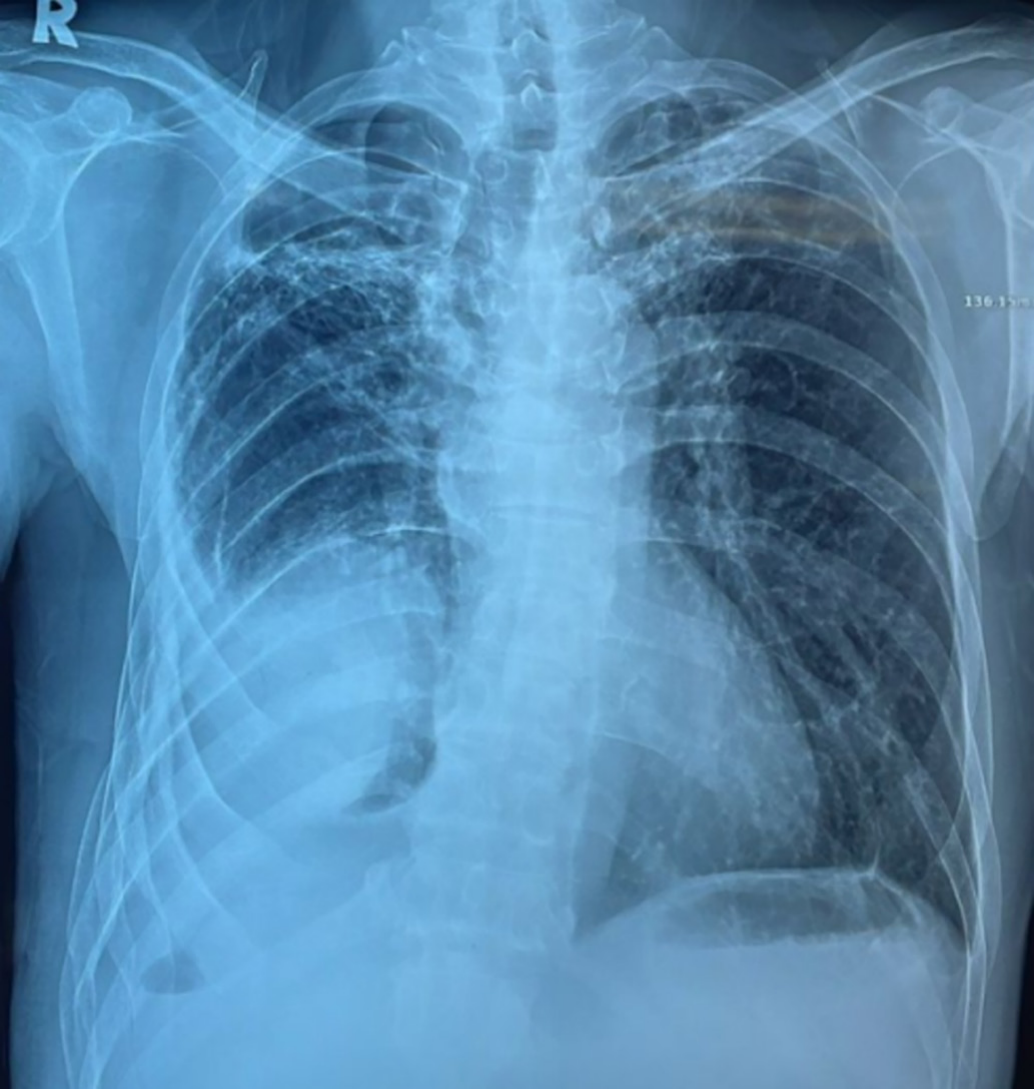
Chest radiograph showing loculated opacity, rib crowding, and right‐sided volume loss.

**FIGURE 3:**
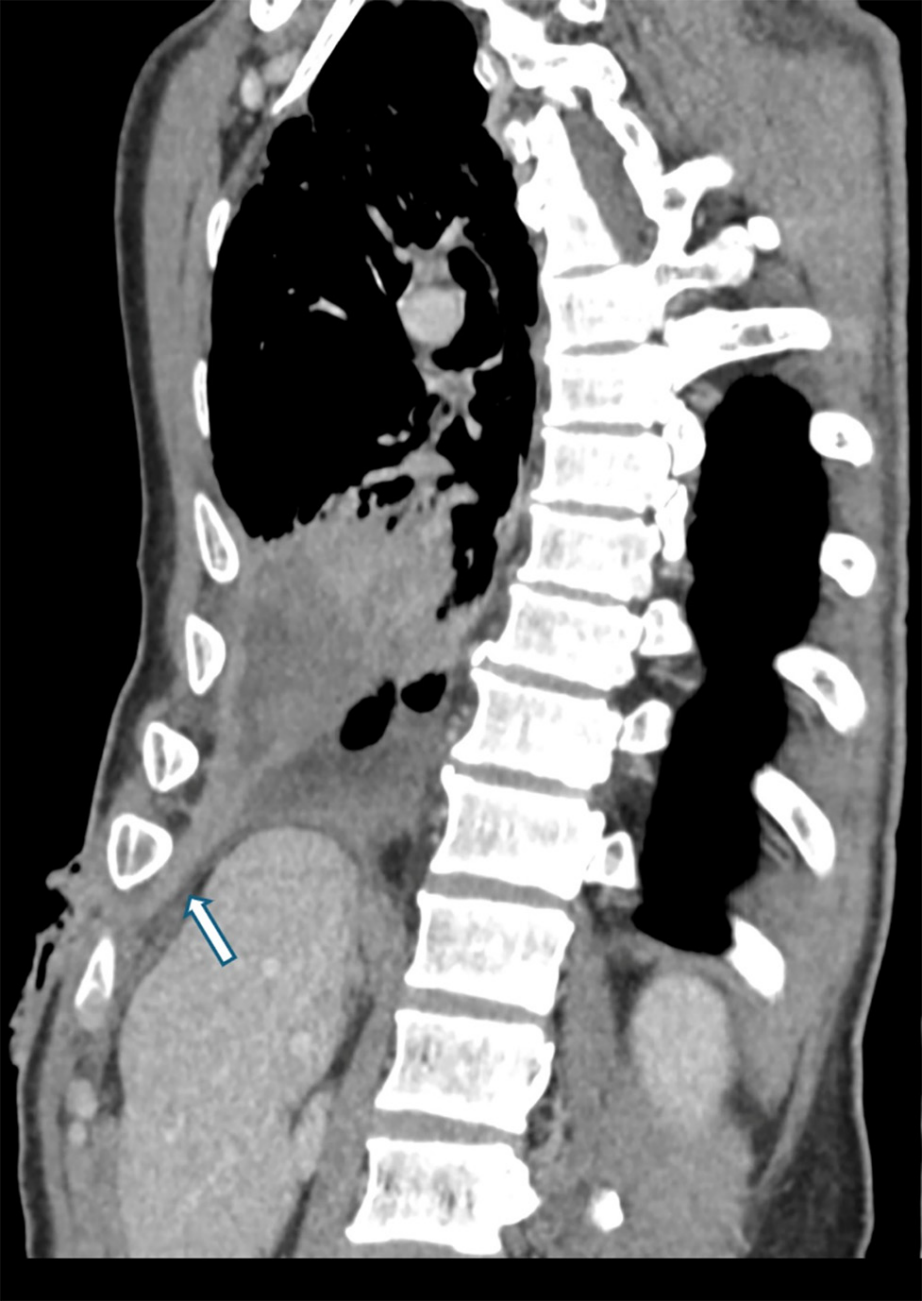
Sagittal CT demonstrating hypodense tract between pleural cavity and skin (arrow).

Despite a full year of treatment, the discharge was persistent. Although initial cultures grew *Klebsiella pneumoniae* , later specimens isolated an extended‐spectrum beta‐lactamase (ESBL)‐producing strain, indicating antimicrobial resistance development. He was treated with intravenous meropenem but remained symptomatic with recurrent subcutaneous abscesses. Imaging consistently showed thickened pleura (Figure 4: white arrow), rib crowding (Figure 4: red arrow), and subcutaneous collections (Figure 5), consistent with chronic, refractory empyema necessitans. Thoracic surgeons recommended an open thoracostomy (pleural window) as definitive source control.

**FIGURE 4:**
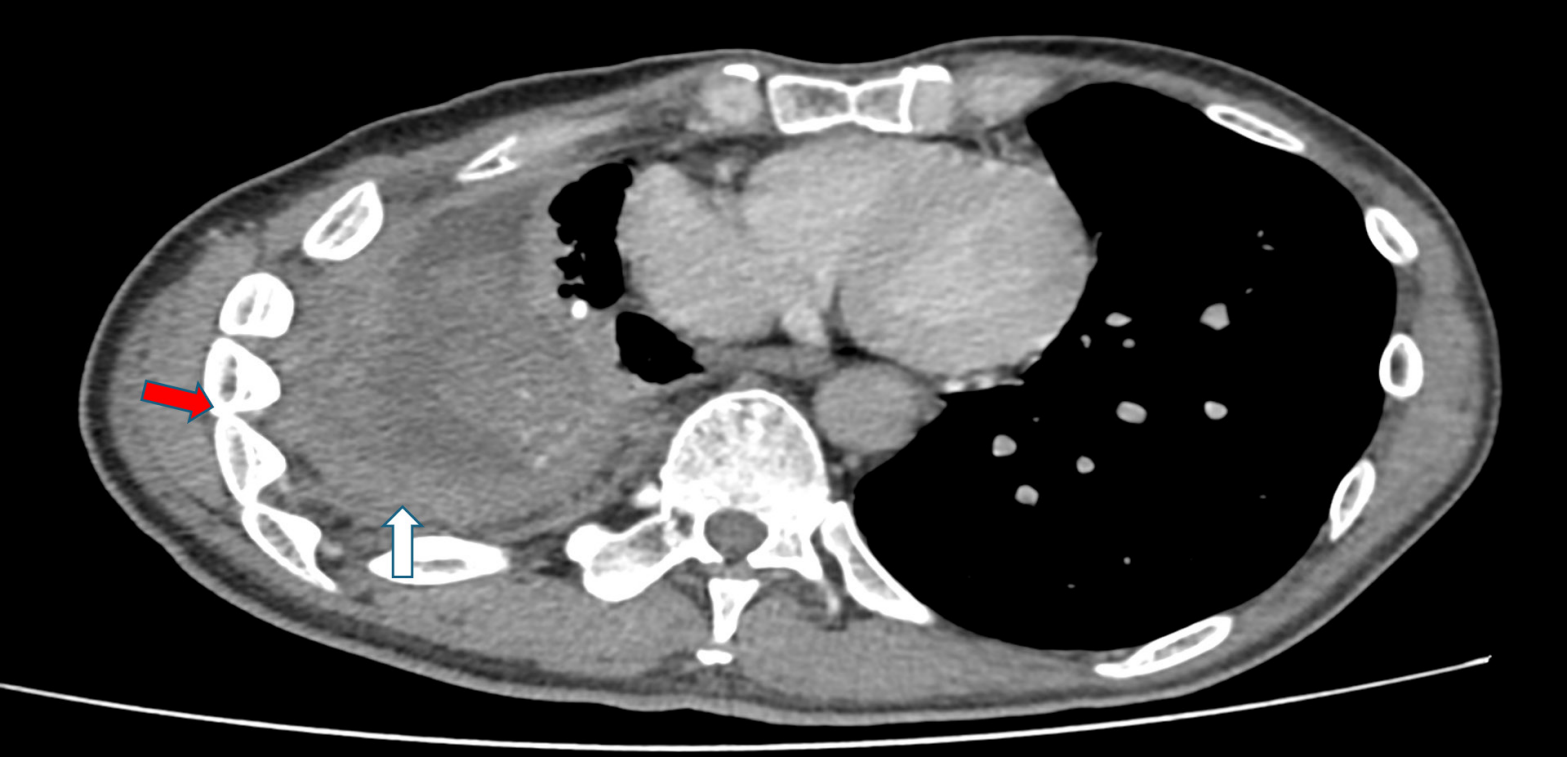
Axial CT showing pleural thickening (white arrow) and rib crowding (red arrow).

**FIGURE 5:**
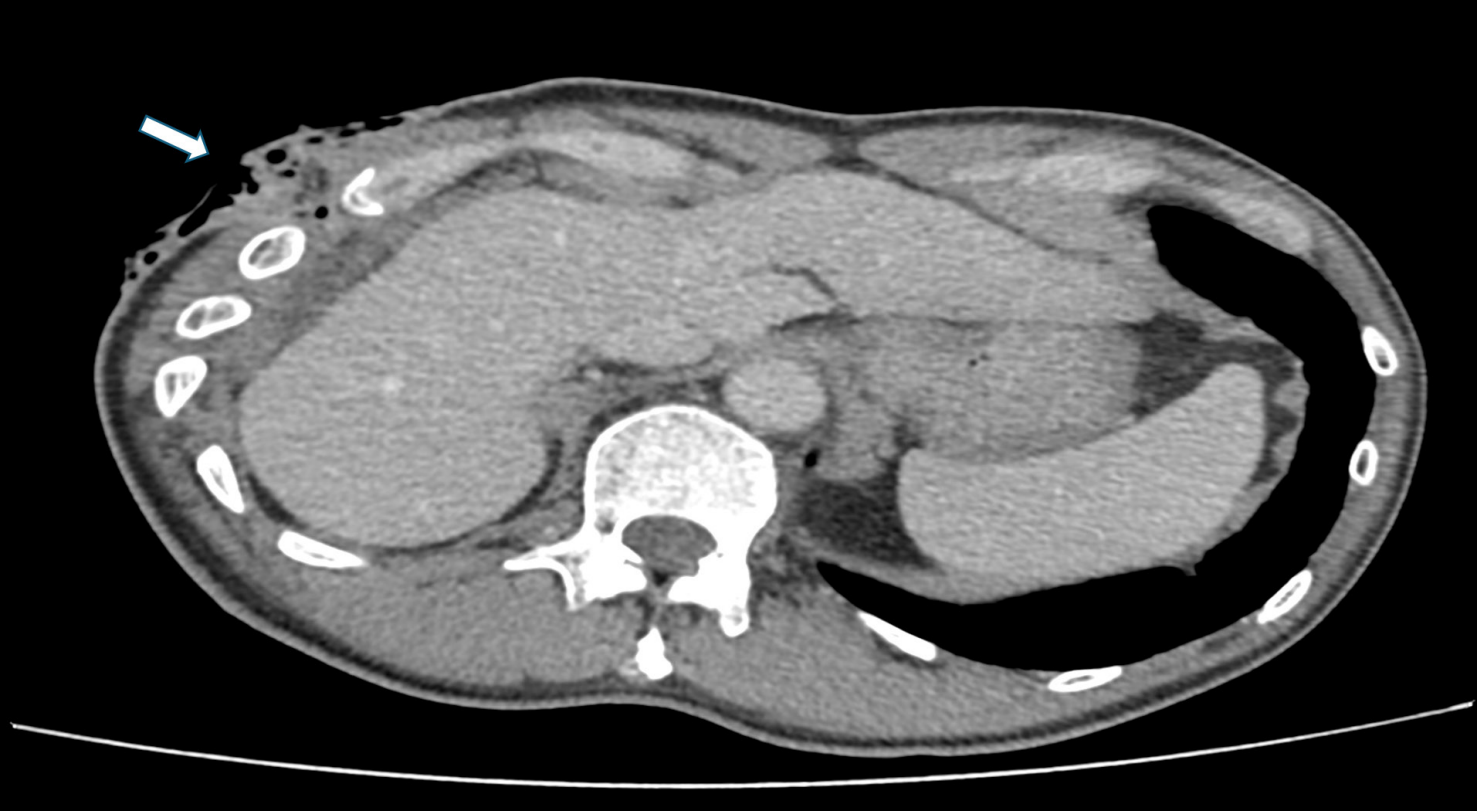
Axial CT depicting subcutaneous collection breaching the right anterior chest wall (arrow).

This case illustrates that not all empyema necessitans are tuberculous; secondary bacterial superinfection (such as *Klebsiella pneumoniae* ) may perpetuate chronic disease [1], resulting in persistently thickened pleura that hampers antibiotic penetration and drainage. Consideration of microbiology and timely source control is thus imperative to avoid progression to refractory, morbid disease, where drastic measures, such as pleural window, may be the only remaining option [2].

## Author Contributions

Zhong Xhen Khor was responsible for data curation, formal analysis, project administration, writing the original draft, and gave final approval.

## Consent

The author declares that written informed consent was obtained for the publication of this manuscript and accompanying images using the consent form provided by the Journal.

## Conflicts of Interest

The author declares no conflicts of interest.

## Data Availability

The data that support the findings of this study are available from the corresponding author upon reasonable request.
